# Rethinking What We Call “Reflux”: A Call for Critical Reappraisal in Brazilian Otolaryngology

**DOI:** 10.1016/j.bjorl.2026.101802

**Published:** 2026-04-13

**Authors:** Adriana Hachiya, Paulo Lins Perazzo, Luciana Miwa Nita Watanabe, André Alencar Araripe Nunes

**Affiliations:** a Faculdade de Medicina da Universidade de São Paulo (FMUSP), São Paulo, SP, Brazil; b Universidade do Estado da Bahia (UNEB), Salvador, BA, Brazil; c Hospital Universitário de Brasília (HUB), Brasília, DF, Brazil; d Universidade Federal do Ceará (UFC), Fortaleza, CE, Brazil

Over the past three decades, patients presenting with laryngopharyngeal symptoms — globus, chronic cough, hoarseness, vocal fatigue, throat clearing, persistent sore throat, foreign body sensation, and thick mucus — have frequently been grouped under a single diagnostic umbrella: laryngopharyngeal reflux (LPR).

In the absence of another evident cause, this label has become commonplace. The publication of the Reflux Finding Score (RFS) in 2001^1^ and, in the following year, the Reflux Symptom Index (RSI)^2^ represented an important milestone by systematizing signs and symptoms attributed to reflux. In Brazil, although attempts were made to apply these scores in clinical practice, their everyday applicability among otorhinolaryngologists proved limited. As a result, terms such as “posterior laryngitis,” reflux laryngitis, or more commonly “signs suggestive of laryngopharyngeal reflux” began to be used. Many of these findings — often isolated and lacking meaningful clinical correlation — have routinely appeared in reports of videolaryngoscopy and flexible nasofibrolaryngoscopy, contributing to an excess of "reflux diagnoses.

Nearly 25 years after the original publication by Koufman and colleagues, which described laryngoscopic findings suggestive of LPR and proposed a widely disseminated scoring system, it is imperative to revisit these concepts in light of more recent scientific advances. The accumulation of evidence over the past two decades demands a critical reassessment of established paradigms, prompting updates in how we approach both the diagnosis and management of these patients.

The outcome of the indiscriminate application of these concepts was predictable: an increase in presumed diagnoses and widespread use of proton pump inhibitors (PPIs), frequently as an empirical therapeutic test and often maintained for months without objective confirmation of disease.

Although some authors consider LPR and gastroesophageal reflux disease (GERD) to be distinct entities with different pathophysiological mechanisms, studies show that up to 50% of patients with LPR symptoms also present typical gastrointestinal symptoms such as heartburn and retrosternal pain.^3^ Conversely, a prevalence of 33–39% of laryngopharyngeal symptoms has been reported among patients with a confirmed diagnosis of GERD.^4^

Flexible nasofibrolaryngoscopy remains a fundamental examination in otorhinolaryngological practice — but its primary role is to exclude other causes rather than to confirm reflux. Edema, hyperemia, interarytenoid thickening, and other alterations are nonspecific. Studies demonstrate that signs classically attributed to reflux can be found in a large proportion of asymptomatic individuals. The RFS itself may be elevated in the absence of objective evidence of reflux and also in conditions such as rhinosinusitis, allergy, or esophageal motility disorders.^5^

Even findings considered more specific, such as granuloma or pseudosulcus, are not pathognomonic. Vocal trauma, vocal fold atrophy, and aging may account for these findings. Nevertheless, the expression “signs suggestive of laryngopharyngeal reflux” continues to appear routinely in reports, often crystallizing a diagnosis that has never been objectively confirmed.

Empirical treatment with PPIs appears simple and safe. However, approximately half of patients with laryngopharyngeal symptoms do not respond adequately to empirical therapy. Diagnostic inaccuracies, non-acid reflux, poor adherence, incorrect medication use, or simply the absence of reflux as the primary cause may explain part of these cases. Despite this, prolonged use of the medication is common, even in the absence of significant clinical improvement. Beyond clinical impact, there are financial costs and potential adverse effects associated with chronic PPI use, including alterations in micronutrient absorption, increased risk of fractures, and possible renal complications. The goal is not to demonize these medications, but rather to use them judiciously and based on sound clinical reasoning.

In this context, the recent San Diego Consensus proposes an important conceptual shift: the term laryngopharyngeal reflux disease should be reserved only for cases with objective evidence of proximal reflux contributing to symptoms occurring at least twice per week for eight weeks or longer. The document emphasizes that endoscopic examinations should not be used as diagnostic methods for reflux and highlights the limitations of the empirical therapeutic test.^6^

Objective investigation — such as pH monitoring or pH-impedance monitoring—is encouraged in selected cases, along with the importance of behavioral and dietary measures.

The consensus also introduces key concepts such as laryngeal hypersensitivity and laryngeal hypervigilance. Hypervigilance refers to excessive attention directed toward the larynx, frequently associated with persistent symptoms even in the absence of an identifiable organic cause. This condition may contribute to the chronicity and exacerbation of symptoms and complicate appropriate clinical management.

In Brazil, data published in the Brazilian Journal of Otorhinolaryngology show that most otorhinolaryngologists base the diagnosis of LPR on a combination of symptoms, endoscopic findings, and response to empirical therapy. In this study, a reflux-related questionnaire was sent to 5,406 otorhinolaryngologists, with responses obtained from only 220 participants.^7^ The results showed that two thirds of Brazilian otorhinolaryngologists based the diagnosis of laryngopharyngeal reflux on the evaluation of both symptoms and clinical findings, in addition to a positive response to empirical therapeutic testing. It is important to emphasize once againt the limitations of the therapeutic test as a diagnostic approach, given the subjectivity of symptoms, the self-limited course of some diseases presenting with laryngopharyngeal symptoms, and the placebo effect, whose efficacy may be comparable to that of proton pump inhibitors. In our country, there is no established routine for diagnostic investigation when empirical therapy fails; instead, PPIs are often continued even without objective evidence of improvement.

Laryngopharyngeal symptoms are common in clinical practice, but they cannot be confined to a single diagnostic label. Recognizing their multifactorial nature is essential to avoid simplistic diagnostic assumptions. Posterior edema is not synonymous with reflux. Throat clearing does not necessarily imply acid-related disease. Persistent dysphonia requires careful investigation — not automatic prescription. pH impedance monitoring, although considered the best available method, is far from being a gold standard. Diagnostic criteria and normal parameters remain under debate, and the role of non-acid reflux still requires further clarification. Knowledge gaps persist—but scientific uncertainty cannot be replaced by fragile convictions.

Despite these limitations, it is urgent that Brazilian otorhinolaryngologists critically reassess our clinical practice regarding laryngopharyngeal symptoms — so common in daily consultations and yet frequently underestimated in their diagnostic complexity. This moment offers an opportunity for Brazilian otorhinolaryngology to lead a more critical and evidence-based discussion on laryngopharyngeal symptoms, aligning clinical practice with emerging scientific evidence.

It is time to abandon automatic reasoning and outdated paradigms and adopt a more critical and thoughtful approach.

Shall we move forward together?

## ORCID ID

Adriana Hachiya: 0009-0003-2566-3660

Paulo Lins Perazzo: 0000-0002-7643-5823

Luciana Miwa Nita Watanabe: 0000-0002-4156-6065

André Alencar Araripe Nunes: 0000-0001-5529-5369

## Declaration of competing interest

The authors declare no conflicts of interest.
